# OsNOMT Contributes to Rice Blast Resistance and Viral Defense and Is Associated with Altered Jasmonate Homeostasis

**DOI:** 10.3390/plants15213259

**Published:** 2026-10-22

**Authors:** Li Jiang, Sisi He, Xuanyu He, Sifan Wang, Linying Li, Xueying Zhang, Guoyan Zhao, Dali Zeng, Gaojie Hong, Yuqing He

**Affiliations:** 1 College of Advanced Agricultural Sciences, Zhejiang A&F University, Hangzhou 311300, Chinadalizeng@126.com (D.Z.); 2 State Key Laboratory for Quality and Safety of Agro-Products, Key Laboratory of Biotechnology in Plant Protection of MARA, Key Laboratory of Green Plant Protection of Zhejiang Province, Institute of Virology and Biotechnology, Zhejiang Academy of Agricultural Sciences, Hangzhou 310021, China17355740916@163.com (S.W.); anran6297@126.com (L.L.); zhangxuey89@163.com (X.Z.); zhaogy@alumni.shanghaitech.edu.cn (G.Z.); 3 College of Life and Environmental Sciences, Hangzhou Normal University, Hangzhou 311121, China; 4 The Zhejiang-UK Joint Laboratory on Soil Health and Agri-Product Safety, Hangzhou 310021, China; 5 Key Laboratory of Traceability for Agricultural Genetically Modified Organisms, Ministry of Agriculture and Rural Affairs, Hangzhou 310021, China

**Keywords:** Sakuranetin, OsNOMT, Jasmonic acid, *Mangnaporthe oryzae*, Southern rice black-streaked dwarf virus

## Abstract

Sakuranetin (SAK), a flavonoid phytoalexin synthesized by naringenin 7-O-methyltransferase (NOMT), contributes to rice immunity. However, the relationship between OsNOMT-dependent SAK accumulation, resistance to fungal and viral pathogens, and jasmonate (JA) homeostasis remains unclear. Here, we combined genetic, metabolite and transcriptomic analyses of *OsNOMT*-overexpression (*OsNOMT-OE*) and knockout (*osnomt*) lines. Loss of *OsNOMT* abolished SAK accumulation and was associated with increased susceptibility to *Magnaporthe oryzae* and Southern rice black-streaked dwarf virus, whereas *OsNOMT-OE* lines accumulated high levels of SAK and exhibited enhanced resistance to both pathogens. Co-expression network analysis identified a susceptibility-associated module containing *OsWRKY62*, *OsFBX148*, and a putative ACC oxidase as candidate hub nodes. *OsNOMT-OE* plants exhibited increased expression of JA-related genes and higher JA levels but lower basal levels of JA-Ile than wild-type ZH11 plants. In *osnomt* mutants, basal JA-Ile levels were reduced, whereas JA-Ile levels after *M. oryzae* infection were comparable to those in ZH11 plants, indicating that *OsNOMT*-dependent SAK accumulation is linked to a reconfiguration of JA homeostasis rather than a uniform increase in all JA metabolites. In addition, short-term exogenous SAK application promoted JA and JA-Ile accumulation. Collectively, these findings associate *OsNOMT*-mediated SAK biosynthesis with rice resistance to fungal and viral diseases and altered JA homeostasis.

## 1. Introduction

Sakuranetin (SAK) is a flavonoid phytoalexin that functions as an important defense-associated metabolite in rice and other plant species. Previous studies have shown that SAK contributes to resistance against diverse biotic stresses, including the fungal pathogen *Magnaporthe oryzae*, *Botrytis cinerea*, *Colletotrichum species*, the bacterial pathogen *Xanthomonas oryzae* pv. *oryzae* (Xoo), and insect herbivory by the brown planthopper [1,2,3,4,5,6]. Beyond its function in plant immunity, SAK also exhibits anticancer, antioxidant, and antimicrobial activities, highlighting its potential therapeutic value [7,8].

The biosynthetic pathway of SAK follows the flavonoid metabolic route. Phenylalanine acts as the starting precursor, which is successively converted into SAK through the catalytic actions of phenylalanine ammonia lyase (PAL), cinnamate-4-hydroxylase (C4H), 4-coumarate:CoA ligase (4CL), chalcone synthase (CHS), chalcone isomerase (CHI), and naringenin 7-O-methyltransferase (NOMT) [9,10,11]. Naringenin 7-O-methyltransferase (NOMT) then catalyzes the O-methylation of naringenin at the 7-position to produce SAK [11,12,13,14]. Because NOMT catalyzes the committed branch step toward SAK formation, it provides a useful entry point for dissecting the contribution of SAK biosynthesis to rice immunity.

SAK accumulation can be induced by various factors, including brassinosteroids (BR), jasmonate (JA), ultraviolet radiation (UV), mechanical wounding, phosphate starvation, and infection by blast fungus [2,11,15]. Several transcription factors, including OsBZR1, OsMYB1R, OsSTK, OsMYC2, OsWRKY67, and OsPHR2 in rice, as well as CsMYC2.1 in tea, have been reported to regulate NOMT promoter activity, thereby modulating SAK accumulation [2,6,16,17,18,19,20]. These findings place NOMT downstream of hormone- and stress-responsive transcriptional networks, particularly JA- and BR-related pathways.

The defense function of SAK is multifaceted. SAK suppresses hyphal growth and spore germination [4,18], inhibits clathrin-mediated endocytosis (CME) of plasma membrane proteins to confer resistance to rice blast [21], and affects insect-associated symbionts required for brown planthopper fitness [3]. However, whether OsNOMT-mediated SAK accumulation is connected with broader immune signaling pathways, and whether this metabolic module contributes to resistance against pathogens with distinct lifestyles, remains insufficiently understood.

The crosstalk between phytohormones and secondary metabolites is central to plant adaptation to developmental and environmental cues, as well as to abiotic and biotic stress [22]. The phytohormones, such as JA, BR, gibberellins (GAs), auxin, cytokinin (CK), Abscisic acid (ABA), salicylic acid (SA), ethylene (ET), and strigolactones (SLs), regulate numerous branches of specialized metabolism [23]. In the case of SAK, JA induces *OsNOMT* expression and SAK accumulation in rice and tea, whereas BR promotes SAK accumulation and basal resistance to *M. oryzae* in rice [2,6,17]. BR enhances SAK accumulation to confer basal resistance to *M. oryzae* [2]. These studies support a model in which JA and other hormones act upstream of SAK biosynthesis.

By contrast, much less is known about the reciprocal direction of this interaction, namely whether perturbation of a specialized metabolic pathway can reshape hormone homeostasis or signaling output. Examples from other systems suggest that such feedback is possible. Disruption of indole glucosinolate biosynthesis in *Arabidopsis* alters auxin accumulation, and overexpression of Rosa rugosa anthocyanidin reductase in tobacco enhances ABA accumulation [24,25,26]. However, whether genetic alteration of *OsNOMT* and consequent changes in SAK accumulation influence JA homeostasis or defense signaling in rice remains unknown.

In rice, *M. oryzae* and Southern rice black-streaked dwarf virus (SRBSDV) represent two biologically distinct disease systems. *M. oryzae* is a hemibiotrophic fungal pathogen that directly invades host tissues [27], whereas SRBSDV is an insect-transmitted viral pathogen whose successful infection depends on host antiviral immunity and vector-mediated transmission [28]. Testing *OsNOMT* function in both pathosystems allows us to evaluate whether *OsNOMT*-dependent SAK accumulation acts only in antifungal defense or instead contributes to a broader immune state that can restrict pathogens with different lifestyles. This distinction is important for assessing the potential of the *OsNOMT*-SAK module in broad-spectrum disease resistance.

To address these questions, we analyzed *OsNOMT*-overexpression and CRISPR/Cas9 knockout rice lines in the ZH11 background using pathogen infection assays, metabolite and hormone quantification, and transcriptome profiling. We examined the effects of altered *OsNOMT* expression on SAK accumulation and responses to *M. oryzae* and SRBSDV and identified associated changes in defense-related co-expression networks and JA-related traits. Our results support a contribution of *OsNOMT* to rice disease resistance and identify changes in JA homeostasis associated with perturbation of the *OsNOMT*–SAK metabolic module. These findings provide a basis for testing whether JA-related processes contribute to the observed resistance phenotypes.

## 2. Results

### 2.1. OsNOMT Positively Regulates Rice Resistance Against M. oryzae

**Figure 1:**
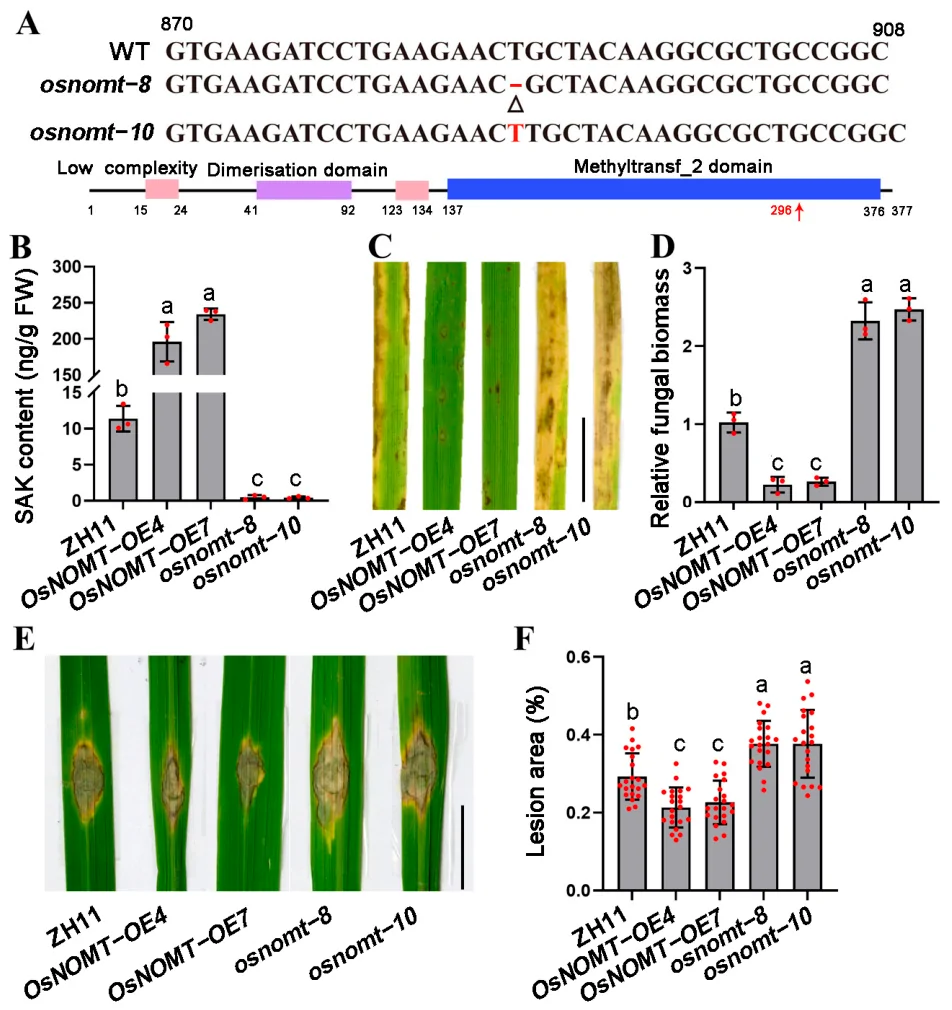
*OsNOMT* positively regulates rice blast resistance. (**A**) Nucleotide sequences at the target site in *osnont* mutant rice plants. The “−” indicates the deleted nucleotide. The red “T” means the inserted nucleotide. The Protein domain was predicted by SMART online (https://smart.embl.de/, accessed on 15 March 2018). (**B**) SAK content in *OsNOMT-OE*, *osnomt*, and ZH11 plants. (**C**,**E**) Blast infection assay in indicated plants. Two-week-old seedlings were sprayed (**C**), whereas four-week-old plants were punch-inoculated (**E**) with blast fungus at 28 °C for five days. (**D**,**F**) Biomass and lesion area of the *M. oryzae*-inoculated leaves in (**C**,**E**), respectively. Different letters indicate significant differences (one-way ANOVA, Fisher’s least significant difference tests test; *p* < 0.05, *n* = 3 for (**B**,**D**), *n* = 20 for (**F**); Bar = 1 cm.

Our previous study reported that overexpression of *OsNOMT* (*OsNOMT-OE*) enhances rice resistance to *M. oryzae* [2]. To further explore the role of *OsNOMT* in plant immunity, we generated two CRISPR/Cas9 knockout mutants, *osnomt-8* and *osnomt-10*, in the ZH11 background (Figure 1A). UPLC-MS analysis revealed that SAK levels were significantly higher in *OsNOMT-OE* plants compared to the wild-type ZH11, while SAK accumulation was nearly undetectable in the *osnomt* mutants (Figure 1B and Figure S1). Following spray inoculation of two-week-old seedlings with *M. oryzae* strain Guy11 spores, *OsNOMT-OE* plants exhibited milder disease symptoms and supported lower fungal biomass than ZH11 plants, whereas *osnomt* mutants showed more severe symptoms and accumulated more fungal biomass (Figure 1C,D). After punch inoculation of four-week-old plants, fungal lesion areas were smaller in *OsNOMT-OE* plants and larger in *osnomt* mutants compared with ZH11 (Figure 1E,F). Together, these results support the contribution of *OsNOMT* to SAK accumulation and resistance to *M. oryzae*.

### 2.2. PCA and K-Means Clustering Analysis

**Figure 2:**
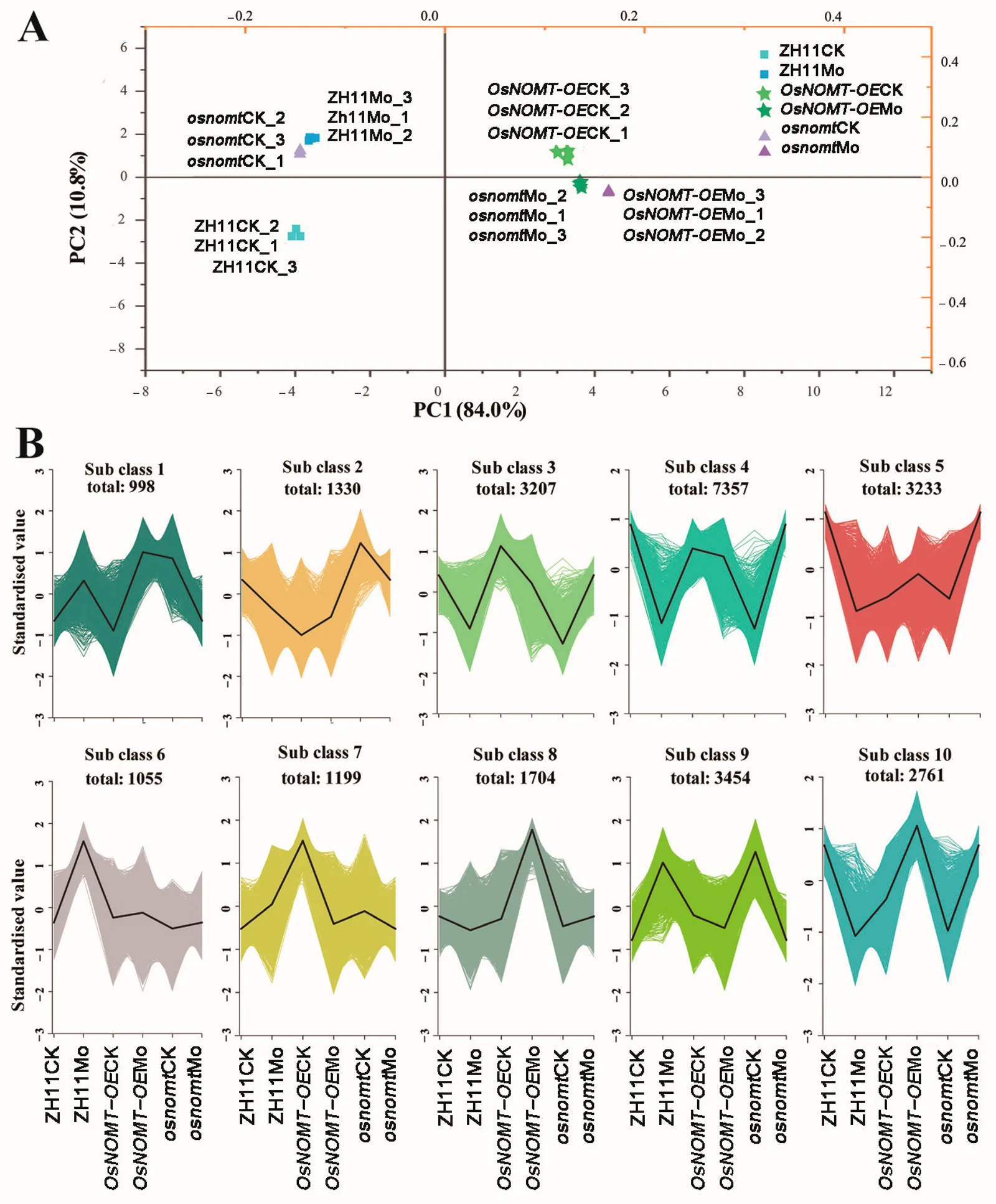
K-means clustering analysis of all annotated genes. (**A**) Sample principal component analysis (PCA). The two mutually perpendicular coordinates at the bottom indicate principal components 1 and 2, respectively. Each variety contains three replicates consisting of at least ten seedlings. (**B**) Expression dynamics of ten co-regulated gene clusters identified by K-means clustering.

To investigate the transcriptional changes associated with altered *OsNOMT* expression, RNA-Seq was utilized to analyze the expression profiles of *M. oryzae*-infected for 24 h and mock-treated ZH11, *OsNOMT*-*OE4*, and *osnomt-8* plants. This process generated 852.49 million raw sequencing reads, amounting to 127.88 Gb of data. Following stringent quality control measures, each cDNA library yielded 41.74–49.58 million high-quality clean reads, accumulating to 124.94 Gb, with an average of 6.94 Gb per library. Quality assessments revealed that more than 95.69% of the bases achieved Phred scores of ≥30. The high-quality clean reads displayed well-balanced base compositions, with GC contents exceeding 52.13% (Supplementary Table S1), indicating that the sequencing data were suitable for downstream analyses. Principal component analysis (PCA) showed clear separation among genotypes and treatments (Figure 2A). PC1 and PC2 explained 84.0% and 10.8% of the total variance, respectively, together accounting for 94.8% of the transcriptomic variation. *OsNOMT-OE* samples under both mock and *M. oryzae*-inoculated conditions clustered toward the high-value region of PC1, whereas ZH11 and *osnomt* samples showed distinct distributions along PC1 and PC2. These patterns indicate that *OsNOMT* expression status and pathogen treatment are major factors contributing to global transcriptomic variation.

To investigate the intricate transcriptional alterations and identify co-regulated patterns, we conducted K-means clustering analysis on all annotated genes, assigning them into 10 distinct modules according to their expression profiles (Figure 2B and Figure S2). Following this, each cluster was analyzed for pathway enrichment using the Kyoto Encyclopedia of Genes and Genomes (KEGG). Genes within subclass 8 showed specific and robust induction in overexpressing plants following pathogen challenge (Figure 2B and Figure S2H, and Supplementary Table S2). These genes were significantly enriched in pathways associated with cutin, wax, and lignin biosynthesis, and phenylpropanoid, starch, and sucrose metabolism. Genes in subclasses 3 and 7 maintained elevated expression levels in healthy leaves of overexpressing plants, even in the absence of inoculation (Figure 2B and Figure S2C,G). They were predominantly involved in RNA splicing, proteasome degradation, RNA transcription, and aminoacyl-tRNA synthesis, which are fundamental cellular functions. Genes in subclass 4 displayed a complex regulatory pattern, with high induction observed in both healthy wild-type plants and inoculated knockout mutants (Figure 2B and Figure S2D). This cluster was highly enriched in pathways related to endoplasmic reticulum protein processing, vesicle transport, and protein glycosylation. These gene expression changes correlate with the observed impairment in protein homeostasis and defense responses in *osnomt* mutants. These clustering results indicate that altered *OsNOMT* expression is associated with broad changes in transcriptional programs related to specialized metabolism, cellular protein homeostasis, and stress responses. In particular, the enrichment of defense-associated metabolic pathways in *OsNOMT-OE* plants after pathogen challenge is consistent with their enhanced resistance phenotype.

### 2.3. Differentially Expressed Genes (DEGs) Analysis

**Figure 3:**
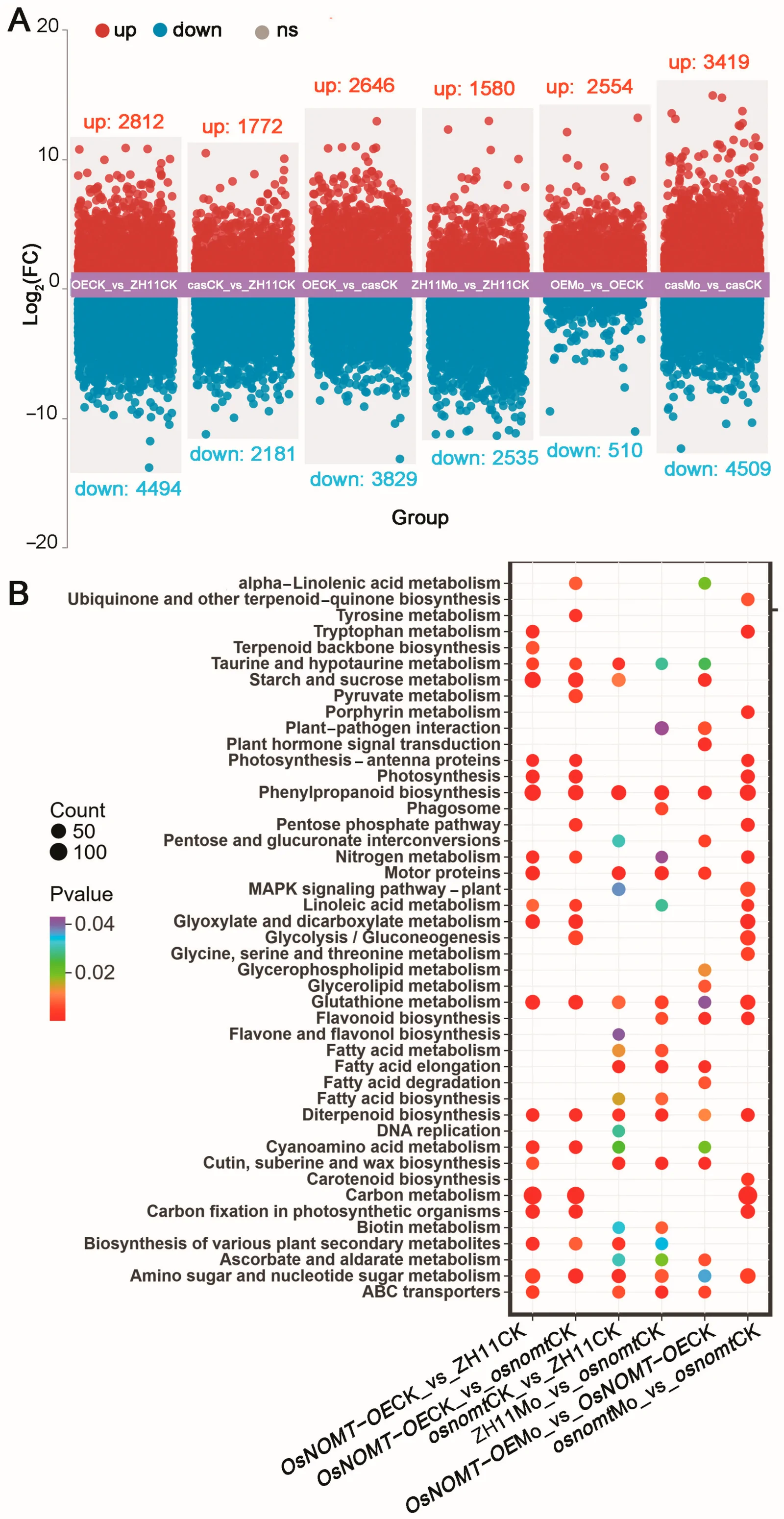
Transcriptome analysis of differentially expressed genes. (**A**) Volcano plot of gene expression differences from *OsNOMT-OE* (OE), *osnomt* (cas), and ZH11 plants, with or without *M. oryzae* inoculation (red dots represent up-regulated genes; blue dots represent down-regulated genes; grey dots represent not significantly expressed genes). (**B**) KEGG analysis of different expressed genes in (**A**). Different genes were enriched with the criteria of |Log_2_ (fold change, FC) | ≥ 1 and *p* < 0.05.

DEGs were identified using the threshold of |log2(fold change, FC)| ≥ 1 and FDR < 0.05. 7306 DEGs (2812 up-regulated and 4494 down-regulated) in the *OsNOMT-OE*CK_vs_ZH11CK comparator group; 4115 DEGS (1580 up-regulated and 2535 down-regulated) in the ZH11Mo vs ZH11CK group; 3064 DEGs (2554 up-regulated and 510 down-regulated) in the *OsNOMT-OE*Mo vs *OsNOMT-OE*CK group; 3953 DEGs (1772 up-regulated and 2181 down-regulated) in the *osnom*tCK vs ZH11CK group; 7928 DEGs (3419 up-regulated and 4509 down-regulated) in the *osnom*tMo vs *osnom*tCK group; and 6475 DEGs (2646 up-regulated and 3829 down-regulated) in the *OsNOMT-OE*CK vs *osnom*tCK group (Figure 3A and Supplementary Table S3). The altered genes were further analyzed in the Kyoto Encyclopedia of Genes and Genomes (KEGG) database, and the top 20 pathways with the highest enrichment levels are listed (Figure 3B, Supplementary Table S3). For each group, KEGG pathways with a *p*-value < 0.05 were further analyzed using aggregation analysis (Figure 3B). The co-enriched pathways were primarily enriched in growth-related primary metabolism (e.g., glutathione, amino sugar, nucleotide sugar, Taurine, hypo-taurine metabolism, nitrogen, starch, and sucrose metabolism) and stress resistance-related secondary metabolism (flavonoid, phenylpropanoid, and diterpenoid biosyntheses) (Figure 3B). Enriched plant hormone signal transduction, as well as linoleic acid and α-Linolenic acid metabolism, were also observed. The MAPK signaling pathway was observed in the *osnomtcas*_CK vs. ZH11_CK group. Because clusters 7 and 8 showed prominent expression changes in *OsNOMT-OE* plants, we performed Venn analyses of the DEGs in the *OsNOMT-OE*CK vs ZH11CK_up and *OsNOMT-O*EMo_vs_*OsNOMT-OE*CK_up groups, identifying 201 commonly up-regulated genes (Figure S3A). KEGG enrichment analysis of these genes revealed significant enrichment in plant–pathogen interactions, the MAPK signaling pathway, diterpenoid biosynthesis, and plant hormone signal transduction (Figure S3B). Together, these data suggested that, in addition to affecting SAK synthesis, alteration of *OsNOMT* expression was associated with broader transcriptional reprogramming of primary and secondary metabolism and hormone-related signaling pathways.

### 2.4. WGCNA Identifies OsNOMT-Associated Co-Expression Modules

**Figure 4:**
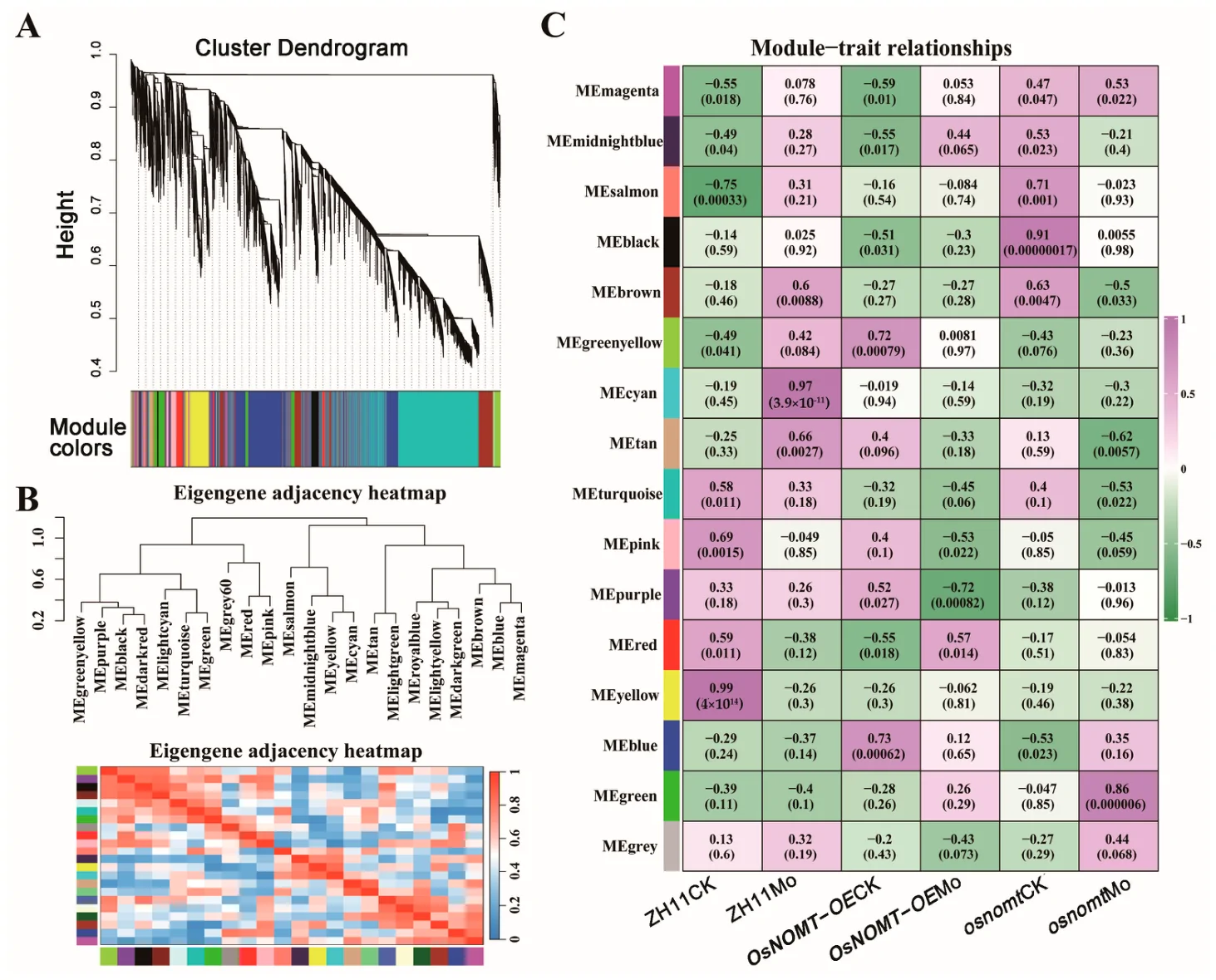
WGCNA identified key modules associated with *OsNOMT*-mediated resistance. (**A**) Gene-clustering dendrogram and module assignment showing the co-expression relationships among all analyzed genes. Each leaf at the end of the tree represents an individual gene, and the color bar below the dendrogram indicates the module assignment of each gene. (**B**) Module eigengene adjacency heatmap showing the pairwise correlations (adjacency) between the eigengenes of all identified modules. (**C**) Module–trait correlation analysis. Each row represents a co-expression module identified by WGCNA, named by a specific color. Each column represents a sample group. The color in each cell indicates the Pearson correlation coefficient between the module eigengene and the corresponding trait. The numbers within each cell represent, from top to bottom, the correlation coefficient and *p*-value, respectively.

To identify gene modules associated with disease resistance and regulated by *OsNOMT*, we performed weighted gene co-expression network analysis (WGCNA). A soft threshold power of 14 was selected, as it yielded an R^2^ > 0.85 for scale-free topology fit, a moderate mean connectivity, and a left-skewed distribution in the connectivity histogram (Figure 4A). These parameters collectively indicate that the data are suitable for constructing a scale-free co-expression network. Hierarchical clustering analysis based on gene expression similarity grouped the genes into thirty-one co-expression modules (Figure 4B), and the presence of multiple distinct and well-defined clustered modules indicates strong functional cohesion within the gene network, while the pronounced color gradient contrast reflects highly interconnected relationships among genes (Figure. 4C). Among them, the MEyellow showed a significantly positive correlation with ZH11CK (cor = 0.99, *p* = 4 × 10^−14^) (Figure 4C and Figure S4A). The MEcyan showed a significantly positive correlation with ZH11Mo (cor = 0.97, *p* = 3.9 × 10^−11^) (Figure 4C and Figure S4B). The MEgreenyellow showed a significantly positive correlation with *OsNOMT-OE*CK (cor = 0.72, *p* = 0.00079) (Figure 4C and Figure S4C). The MEred showed a highly positive correlation with *OsNOMT-OE*Mo (cor = 0.57, *p* = 0.014) (Figure 4C and Figure S4D). The MEbrown showed a significantly positive correlation with *osnomt*CK (cor = 0.63, *p* = 0.0047) (Figure 4C and Figure S4E). The MEgreen showed a significantly positive correlation with *osnomt*Mo (cor = 0.86, *p* = 0.000006) (Figure 4C and Figure S4F). These results identify co-expression modules associated with *OsNOMT* expression and pathogen treatment, providing a framework for prioritizing candidate defense-related genes.

### 2.5. Candidate Hub Genes in the Osnomt Susceptibility-Associated Module

**Figure 5:**
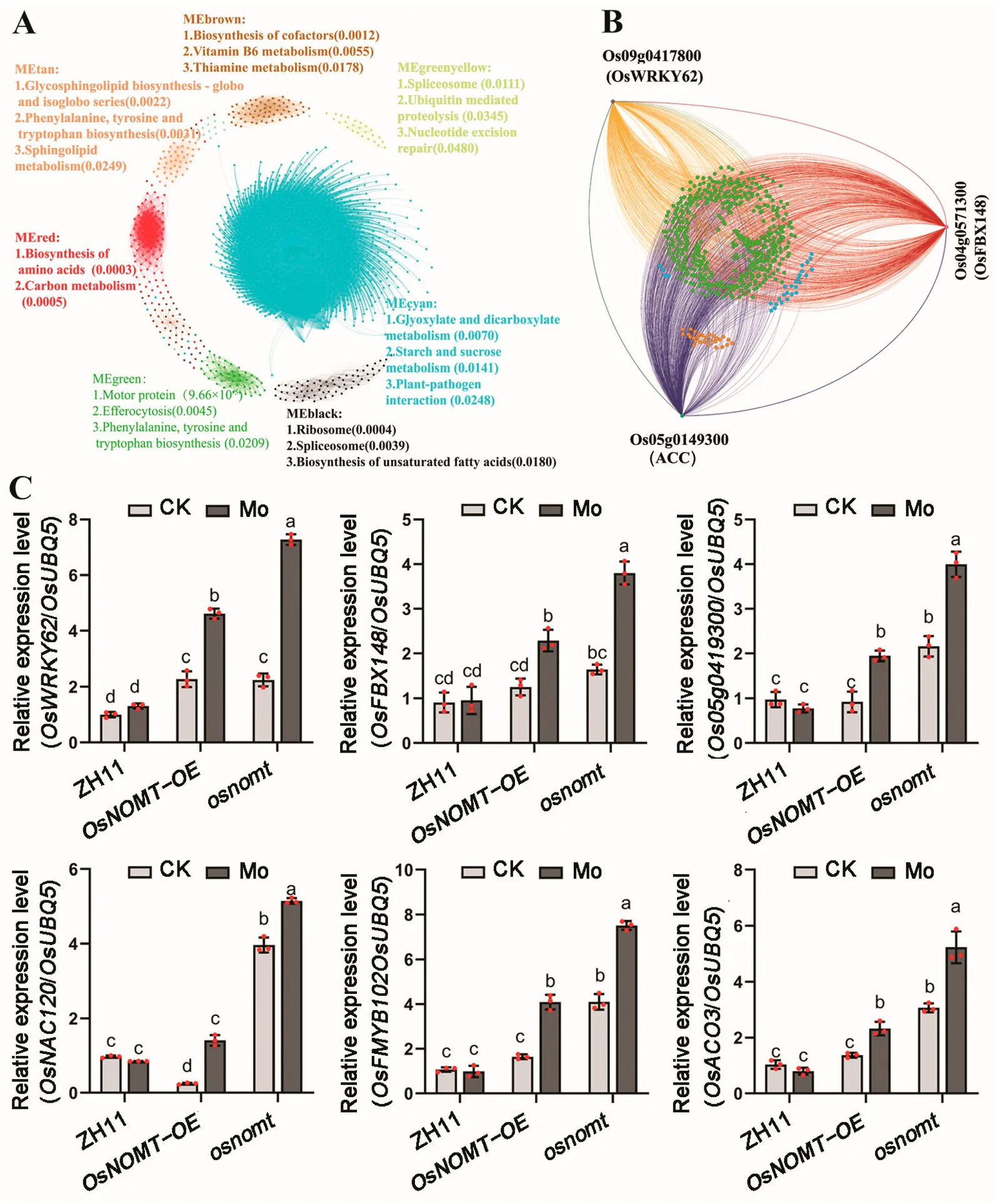
*OsNOMT*-mediated defense model and key regulatory hubs. (**A**) Functional allocation of co-expression modules dominated by *OsNOMT* under different conditions. Data from the WGCNA module-trait correlation analysis in Figure 4C. Each colored sphere represents a gene co-expression module, positioned according to its most associated condition (r > |0.8|, *p* < 0.01). Key KEGG pathways significantly enriched in each module are labeled next to the corresponding sphere. (**B**) Regulatory network and hub genes identified from genes significantly up-regulated in *osnomt* mutants after infection with *M. oryzae*. Three hub genes were *OsWRKY62*, *OsFBX148*, and *Os05g0149300* (ACC). Orange nodes represent genes connected to only one hub gene, blue nodes represent genes connected to two hub genes, and green nodes represent genes connected to three hub genes. (**C**) RT-qPCR verification of the differentially expressed genes in B. Different letters indicate significant differences (ANOVA, Fisher’s least significant difference test; *p* < 0.05, *n* = 3).

To prioritize candidate genes within genotype- and treatment-associated modules, we utilized module membership-gene significance (MM-GS) scatter plots to prioritize hub genes (Figure 5A and Supplementary Table S4). KEGG enrichment of the module gene sets indicated functional specificity across genotypes and treatments. In ZH11, distinct modules were associated with basal growth and innate defense (Figure 5A and Figure S5A,B). In *OsNOMT-OE* plants, modules associated with mock-treated samples were enriched in protein export, tRNA biosynthesis, RNA splicing, and ubiquitin-mediated proteolysis, whereas the module associated with *M. oryzae*-inoculated *OsNOMT-OE* plants was enriched in carbon metabolism, energy-related pathways, and flavonoid biosynthesis (Figure 5A and Figure S5C–E). In *osnomt* mutants, modules associated with mock-treated and M. oryzae-inoculated samples were enriched in ribosome biogenesis, RNA splicing, and amino acid biosynthesis-related pathways (Figure 5A and Figure S5F,G). These results suggest that *OsNOMT* overexpression and loss of *OsNOMT* are associated with distinct co-expression programs during pathogen challenge.

We next focused on the MEgreen module, which was strongly associated with *M. oryzae*-inoculated *osnomt* mutants, to identify highly connected candidate genes. Three genes, *OsWRKY62* (*Os09g0417800*), OsFBX148 (*Os04g0571300*), and a putative ACC oxidase (Os05g0149300), showed high intramodular connectivity and were selected as candidate hub genes (Figure 5B and Table S5). These genes showed significantly elevated baseline expression in *osnomt* mutants relative to the ZH11 control and remained highly expressed following *M. oryzae* infection (Figure 5B). RT–qPCR validation confirmed the transcriptomic patterns (Figure 5C). *OsWRKY62* has been reported to negatively regulate resistance to *M. oryzae* and *Xoo* [29]. *OsFBX148*, a component of the SKP1-CUL1-F-box protein (SCF) ubiquitin ligase complexes, negatively regulates ABA-mediated drought stress responses [30]. Predicted ACC oxidase (*Os05g0149300*) is potentially involved in ethylene synthesis. Therefore, the elevated expression of these candidate hub genes in *osnomt* mutants is consistent with a susceptibility-associated transcriptional state, although their direct regulatory relationships with *OsNOMT* or SAK remain to be determined.

### 2.6. Altered OsNOMT Expression Is Associated with Changes in JA-Related Gene Expression and Hormone Levels

**Figure 6:**
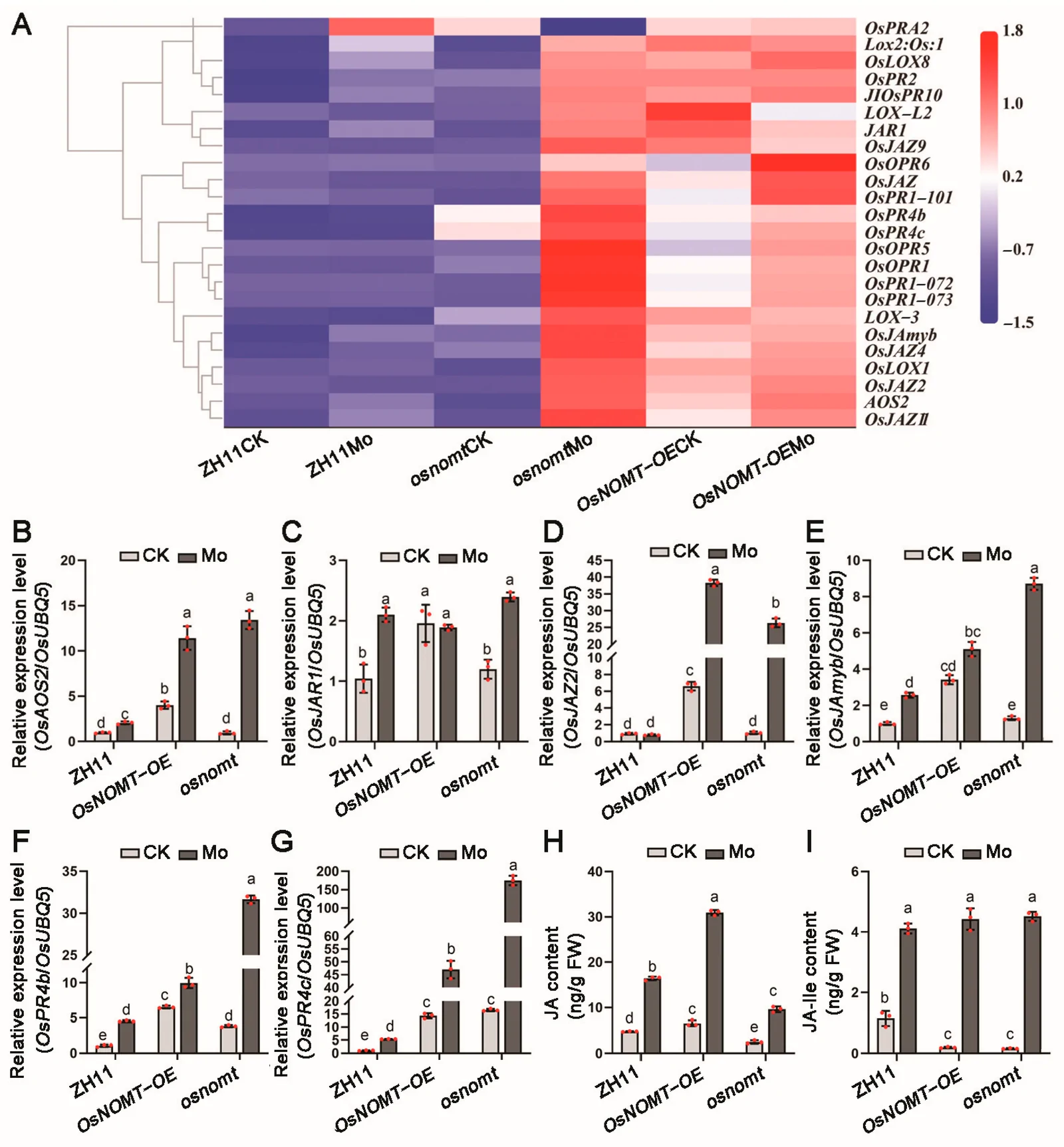
JA-related gene expression and JA and JA-Ile levels in *OsNOMT* genetic lines. (**A**) Hierarchical cluster heatmap analysis of JA-related genes. (**B**–**G**) RT-qPCR verification of JA-related genes. *OsUBQ5* was set as an internal reference. (**H**,**I**) JA and JA- Ile contents in indicated plants inoculated with or without *M. oryzae* at 24 h. Different letters indicate significant differences at *p* < 0.05 using one-way ANOVA followed by Fisher’s least significant difference test, *n* = 3.

Because α-linolenic acid is the precursor of JA biosynthesis and JA plays a crucial role in the defense against rice blast disease [2], we examined JA-related changes in the OsNOMT genetic lines. KEGG enrichment analysis of DEGs identified linoleic acid metabolism (ko00591) and the α-linolenic acid pathway (ko00592) among the enriched pathways (Figure 3), prompting further analysis of JA-related gene expression and hormone levels. Heatmap clustering analysis further showed that genes involved in JA biosynthesis, signaling, and response were up-regulated in OsNOMT-overexpressing plants compared to the wild-type ZH11, while these genes were down-regulated or unchanged in the *osnomt* mutant (Figure 6A). RT-qPCR analysis of representative genes, OsAOS2, OsJAR1, OsJAmyb, OsJAZ2, OsPR4b, and OsPR4c, supported the expression patterns observed in the transcriptome data (Figure 6B–G). These results indicate that OsNOMT overexpression is associated with enhanced expression of JA-related genes.

We next measured JA and JA- Ile in different genetic backgrounds. JA levels were higher in *OsNOMT-OE* plants and lower in the *osnomt* mutants compared to the wild-type ZH11 (Figure 6H). However, JA-Ile did not change in parallel with JA (Figure 6I). Under basal conditions, *OsNOMT-OE* plants accumulated more JA but less JA-Ile than ZH11 plants, despite their higher SAK and JA levels. The *osnomt* mutants also showed reduced basal JA-Ile levels, whereas their JA-Ile levels were not significantly different from those of ZH11 plants after *M. oryzae* infection for 24 h. Thus, JA abundance did not consistently predict JA-Ile abundance in these genetic backgrounds.

To further investigate whether exogenous SAK application is associated with JA responses, two-week-old plants were sprayed with 0.5 mM SAK or the corresponding mock solution. Quantification of SAK in the treated tissues confirmed that SAK was present at higher levels in SAK-treated plants than in mock-treated plants (Figure S6A). SAK treatment induced the expression of JA-related genes at 1 and 3 h after treatment (Figure S6B). After treatment, at 24 h, both JA and JA-Ile levels were increased in SAK-treated plants compared with mock-treated plants (Figure S6C). These results indicate that acute exogenous SAK application is associated with early activation of JA-related transcription and subsequent JA accumulation under the conditions tested. These results together indicate that altered *OsNOMT* expression is associated with changes in JA-related gene expression and JA homeostasis.

### 2.7. OsNOMT Overexpression Is Associated with Reduced SRBSDV Accumulation

**Figure 7:**
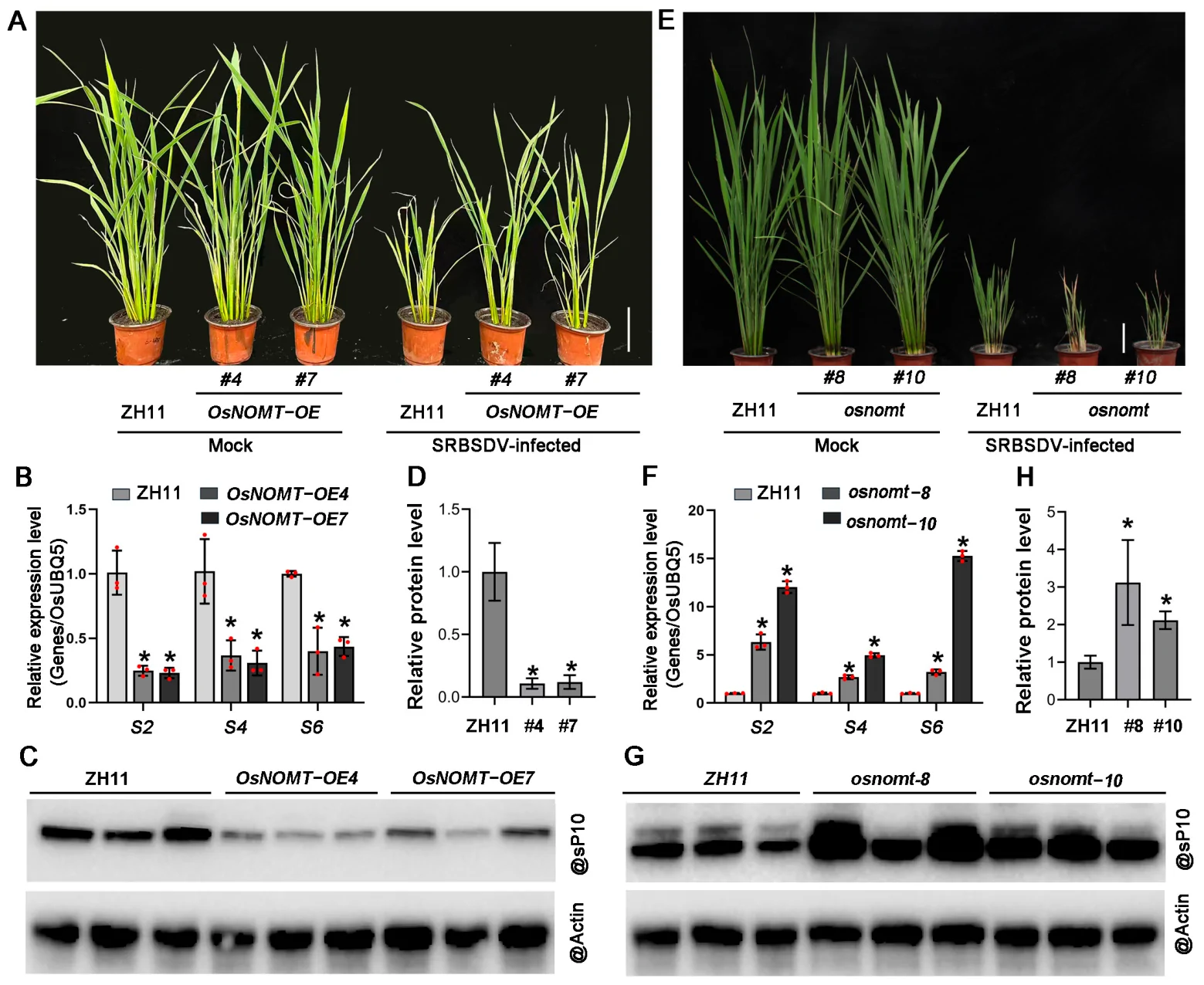
Overexpression of *OsNOMT* promoted rice resistance to SRBSDV. (**A**–**D**) Comparison of *OsNOMT-OE* and ZH11 plants. (**D**–**F**) Comparison of *osnomt* mutants and ZH11 plants. (**A**,**E**) Symptoms in ZH11, *OsNOMT-OE* (#4 and #7), and *osnomt* (#8 and #10) lines, with or without SRBSDV inoculation. Scale bar  =  6 cm. (**B**,**F**) RT-qPCR results showing the relative expression of viral RNA (three different SRBSDV genomic RNA segments *S2*, *S4*, and *S6*). *OsUBQ5* was set as the internal reference gene. (**C**,**G**) Western blot analyzed the accumulation of SRBSDV P10. Actin was used as a loading control. (**D**,**H**) Quantification analyses of (**C**,**G**), respectively. For each viral RNA segment and relative protein level, the indicated line was compared separately with ZH11 using Student’s *t*-test. * means significantly difference at *p* < 0.05, *n* = 3.

To examine whether the *OsNOMT*-associated defense phenotype extends beyond fungal disease, we tested the response of *OsNOMT-OE* plants to SRBSDV, an insect-transmitted rice virus [31]. We inoculated *OsNOMT-OE* lines and wild-type ZH11 plants with SRBSDV-infected or virus-free insect vectors. Following approximately 30 days of growth post-inoculation (dpi) under field conditions, we observed that SRBSDV-infected ZH11 plants manifested typical symptoms such as stunted growth, dark green leaves, rigidity, and excessive tillering in wild-type plants. In contrast, these symptoms were less severe in *OsNOMT-OE* plants (Figure 7A). RT-qPCR analysis revealed that the accumulation of SRBSDV RNA (S2, S4, and S6) was significantly reduced in *OsNOMT-OE* plants compared to ZH11 plants (Figure 7B). The SRBSDV protein (P10) signal was weaker in *OsNOMT-OE* plants than in ZH11 plants in the representative Western blot (Figure 7C,D). In contrast, the *osnomt* mutants were more susceptible to SRBSDV infection (Figure 7E–G). These results indicate that *OsNOMT* overexpression is associated with enhanced resistance to SRBSDV infection, suggesting that the *OsNOMT*-dependent defense phenotype is not limited to *M. oryzae*.

## 3. Discussion

In this study, we conducted transcriptomic analyses to examine how OsNOMT-dependent SAK biosynthesis is associated with rice immunity. PCA and K-means clustering analysis reveal distinct transcriptional profiles among *OsNOMT-OE*, *osnomt*, and wild-type plants under control and *M. oryzae*-inoculated conditions (Figure 2). In *OsNOMT-OE* plants, pathogen challenge was associated with stronger induction of genes related to phenylpropanoid/flavonoid metabolism, cutin, wax and lignin biosynthesis, and carbohydrate metabolism (cluster 3 and 8). These processes have established roles in plant defense [22]. By contrast, *osnomt* mutants showed transcriptional patterns enriched in protein processing, vesicle transport, and basic biosynthetic pathways (cluster 4), indicating differences in cellular responses to *OsNOMT* loss and pathogen challenge. DEGs and KEGG enrichment analysis further indicated that changes in *OsNOMT* expression were associated not only with SAK biosynthesis but also with broader metabolic and hormone-related pathways, including JA-associated processes and the MAPK pathway (Figure 3). Together, these findings associate altered *OsNOMT* expression and SAK accumulation with broader transcriptional changes in defense-related processes. However, they do not establish the direction of these relationships or distinguish responses to altered SAK accumulation from other effects of OsNOMT perturbation.

WGCNA identified a co-expression module in the *osnomt* mutant background, with *OsWRKY62*, *OsFBX148*, and a putative ACC oxidase gene among the most highly connected genes (Figure 5). The reported or predicted functions of these candidate hub genes suggest potential links to defense and stress responses. *OsWRKY62* has been reported to negatively regulate rice resistance to *M. oryzae* and *Xoo* [29,32]. OsFBX148 encodes an F-box protein implicated in ABA-related stress responses [30,33]. Putative ACC oxidase may be involved in ethylene biosynthesis, which participates in plant–pathogen interactions [34,35,36]. These genes exhibited higher basal expression in *osnomt* mutants than in ZH11 and remained highly expressed following *M. oryzae* infection (Figure 5). Their expression patterns, together with an increased susceptibility of the mutants, identify candidate associations for further investigation. However, these observations do not distinguish whether the expression changes contribute to susceptibility, represent compensatory responses, or reflect other physiological changes associated with *OsNOMT* loss. The network analysis therefore provides candidates for functional investigation, while their regulatory relationships with *OsNOMT*-dependent SAK accumulation and their contributions to disease resistance remain to be established.

Previous studies established that JA acts upstream of SAK biosynthesis by inducing *OsNOMT* expression and promoting SAK accumulation through JA-responsive transcriptional regulators such as OsMYC2 [2,17]. BR signaling can also promote *OsNOMT* expression and SAK accumulation through OsBZR1-dependent regulation [2]. These findings place SAK biosynthesis within a broader hormone-responsive defense network. In this work, altered *OsNOMT* expression was associated with changes in JA-related gene expression and JA accumulation. *OsNOMT-OE* plants accumulated higher JA levels and showed altered expression of genes involved in JA biosynthesis, signaling, and defense responses (Figure 6). Short-term exogenous SAK treatment also increased JA and JA-Ile levels (Figure S6). Together, these results raise the possibility that SAK accumulation can influence JA homeostasis or JA-related transcriptional responses. However, they do not establish a direct feedback pathway from SAK to JA signaling. Similar interactions between specialized metabolism and hormone homeostasis have been reported in other plant systems [37,38]. For example, disruption of indole glucosinolate biosynthesis in *Arabidopsis* alters auxin accumulation, and modification of flavonoid or anthocyanin metabolism can affect auxin- or ABA-related processes [24,25,39]. Whether *OsNOMT*-dependent SAK accumulation directly regulates JA metabolism or signaling needs future investigation.

A key feature of our results is that JA and JA-Ile did not change in parallel. *OsNOMT-OE* plants accumulated higher basal JA levels but lower JA-Ile levels than ZH11 plants, whereas *osnomt* mutants showed reduced basal JA-Ile levels but no significant difference from ZH11 after *M. oryzae* inoculation (Figure 6). JA-Ile abundance depends on several processes in addition to JA production, including conjugation of JA to isoleucine, transport, turnover, oxidation, and deconjugation [40]. OsJAR1 catalyzes the formation of JA-Ile from JA and is therefore one possible point at which JA and JA-Ile levels may become uncoupled. OsJAR1 catalyzes the conjugation of JA to isoleucine to generate JA-Ile, whereas JA-Ile can be inactivated through oxidation or deconjugation, and JA signaling is subject to strong feedback through JAZ repressors and other pathway components [41]. The higher OsJAZ2 transcript abundance detected in *OsNOMT-OE* plants (Figure 6D) is consistent with altered expression of a JA-responsive feedback component, but transcript abundance alone does not establish increased JAZ repressor activity or reduced JA-Ile signaling. Thus, one possible explanation for the higher JA and lower basal JA-Ile levels in *OsNOMT-OE* plants is that *OsNOMT* overexpression is associated with changes in JA-Ile conjugation or turnover. Therefore, an increase in JA does not necessarily lead to a proportional increase in JA-Ile.

The response of *osnomt* mutants to *M. oryzae* infection further indicates that JA-related transcriptional responses are dependent on physiological context and sampling time. Although basal JA and JA-Ile levels were reduced in the mutant background, infection induced a subset of JA-related genes, and JA-Ile levels in infected *osnomt* mutants were not significantly different from those in ZH11 (Figure 6). This infection-associated response may reflect pathogen-triggered immune compensation rather than restoration of an effective *OsNOMT*-dependent defense state [42]. Similar observations have been reported for JA-deficient or JA-Ile-related rice mutants, which remain susceptible to blast disease while retaining the capacity to induce subsets of JA-responsive genes after infection or environmental stimulation [41,43,44]. MAPK-mediated pattern-triggered immunity can also stimulate JA biosynthesis and defense gene expression independently of SAK accumulation [18,45,46]. Therefore, basal and pathogen-induced JA responses should not be conflated. Together, the loss of *OsNOMT* was associated with altered basal JA homeostasis and increased susceptibility to *M. oryzae*, whereas pathogen infection induced JA-related responses that were not accompanied by a detectable difference in JA-Ile levels between *osnomt* and ZH11. The persistence of enhanced susceptibility in *osnomt* mutants suggests that infection-induced JA responses did not fully compensate for the loss of *OsNOMT*-dependent SAK accumulation. However, genetic perturbation of JA-Ile biosynthesis or perception will be required to determine whether the resistance phenotype is mediated by altered JA-Ile signaling.

The inclusion of both *M. oryzae* and SRBSDV allowed us to examine whether the disease-associated phenotype of *OsNOMT-OE* plants extends beyond antifungal defense to a pathogen with a distinct lifestyle. For rice blast, SAK may contribute through relatively direct antifungal mechanisms, including inhibition of fungal growth, spore germination, and CME-associated infection processes [4,13,18,19,21]. By contrast, the mechanism underlying reduced SRBSDV accumulation observed in *OsNOMT-OE* plants is likely to be more complex. SRBSDV is transmitted by white-backed planthoppers and replicates in rice as a viral pathogen [31]. Therefore, *OsNOMT*-mediated resistance could result from effects on host antiviral immunity, plant physiological status, vector-mediated inoculation or transmission, viral multiplication, or a combination of these processes.

Our current data do not distinguish among these possibilities. However, *OsNOMT-OE* plants displayed increased expression of JA-related genes and high JA levels (Figure 6). JA signaling has been implicated in rice defense against viral infection and insect-associated stress [3,47]. These observations identify JA-associated defense as a possible factor in the *OsNOMT-OE* phenotype. Future work needs to distinguish whether that SAK directly inhibits SRBSDV infection or replication or through whether JA signaling mediates reduced SRBSDV accumulation. Nevertheless, our data showed that *OsNOMT*-dependent SAK accumulation may be associated with resistance in more than one pathosystem.

*OsNOMT* belongs to the broader O-methyltransferase family, whose members often participate in specialized metabolism and stress adaptation [14]. Recent pan-genome analysis of the O-methyltransferase gene family in tomato revealed extensive evolutionary dynamics and functional divergence among family members, providing a useful framework for understanding how methyltransferases acquire lineage-specific metabolic and defense functions [48]. In this context, *OsNOMT* may represent a rice-specialized methyltransferase that links flavonoid-derived phytoalexin biosynthesis with immune-related signaling outputs. Comparative genomic and biochemical studies of NOMT homologs across rice germplasm and related species will help determine whether variations in *OsNOMT* contributes to natural diversity in SAK accumulation and disease resistance.

## 4. Material and Methods

### 4.1. Plant Material and Rice Blast Inoculation

*OsNOMT* over-expressing (*OsNOMT-OE*) lines and its wild-type *Oryza sativa* L. *japonica var.* Zhonghua 11 (ZH11) are as described [2]. The *osnomt* mutants under ZH11 were obtained from Biogle Genetech (www.seedseek.cn, Hangzhou, China). Pathogen resistance was tested using the *M. oryzae* guy11 strain [19]. The fungus was cultured for two weeks at 25 °C, then washed, adjusted to 1 × 10^5^ or 10^6^ spores/mL in sterile water with 0.05% Tween-20 for the spraying and punching methods, respectively. Two-week-old and one-month-old plants were sprayed or punch-inoculated with the spore suspension, respectively. After inoculation, plants were kept in a 28 °C growth chamber with 85% humidity, dark for 36 h, and then under a 14 h light/10 h dark cycle. Disease symptoms were evaluated with ImageJ (version 1.52a) 5–7 days later. Relative fungal growth was assessed by qPCR, comparing the CT values of *M. oryzae* 28S rDNA to rice *OsACTIN1*. The primers used are listed in Table S6. For exogenous SAK treatment, a stock solution of SAK (Sigma-aldrich, Shanghai, China) was made in absolute ethanol and stored at −20 °C as described [20]. Before treatment, it was freshly diluted with sterile distilled water containing 0.1% Triton X-100. Two-week-old plants were sprayed with 0.5 mM SAK or the corresponding mock solution. The same volume of ethanol containing 0.1% Triton X-100 was used as the mock control. An equal volume of solution was applied to each treatment. Samples were collected at 1 and 3 h after treatment for RNA extraction, and at 24 h for SAK, JA, and JA-Ile determination.

### 4.2. Quantitative Real-Time PCR Analysis

Quantitative real-time PCR (RT-qPCR) was employed to examine the changes in mRNA levels, as previously detailed [19]. Two-week-old seedlings treated with or without *M. oryzae* for 24 h were divided into three biological replicates, with each replicate comprising ten pooled plants. A total of 1 μg of total RNA was reverse-transcribed into first-strand cDNA. RT-qPCR was conducted using the 2X LEYIBIO SYBR qPCR Master Mix (LEYIBIO, Shanghai, China) on a QuantStudio™6 Flex Real-Time PCR System (Applied Biosystems, Singapore) following the manufacturer’s protocol. Thermal cycling conditions were set at 95 °C for 3 min; 95 °C for 15 s, 60 °C for 15 s, and 72 °C for 20 s, for 40 cycles. Melt curve analysis was performed post-amplification by incrementally raising the temperature from 60 °C to 95 °C (0.1 °C/s), with real-time fluorescence acquisition throughout the heating process. The resulting profiles demonstrated exclusive detection of target-specific amplicons, as evidenced by discrete melting peaks. The reference gene *OsUBQ5* was utilized as an internal control, and the relative expression levels were calculated using the comparative C_T_ method (2^−∆∆Ct^). The experiments were performed with a minimum of three biological replicates. The primers used are listed in Table S6.

### 4.3. SAK, JA and JA-Ile Determination

The procedure for the SAK, JA, and JA-Ile measurements was adapted from a previous study [19]. Two-week-old seedlings treated with or without *M. oryzae* for 24 h were collected and finely ground in liquid nitrogen. Subsequently, 100 mg of the ground sample was immersed in 2 mL of an extraction buffer composed of ethanol, water, methanol, and acetic acid in a ratio of 79:13.99:7:0.01 (*v*/*v*/*v*/*v*) and allowed to incubate for 24 h at 4 °C. Following incubation, the mixture was centrifuged at 16,000× *g* for 15 min. The resulting supernatant was then subjected to analysis using UPLC-MS/MS (SCIEX, Framingham, MA, USA, 5500+). The experiment included three biological replicates, with each replicate comprising ten pooled plants.

### 4.4. RNA-Seq Library Preparation and Sequencing

RNA-seq library construction and sequencing were performed by Novogene (https://magic-plus.novogene.com/#/, Beijing, China). Two-week-old ZH11, *OsNOMT-OE4*, and *osnomt-8* plants were treated with sterile water or inoculated with *M. oryzae* for 24 h. Three biological replicates, with each replicate comprising ten pooled plants, were analyzed for each genotype-treatment combination, yielding 18 libraries in total. Libraries were sequenced on the DNBSEQ-T7 platform to generate 150 bp paired-end reads.

Raw reads were filtered using fastp version 0.19.7 to remove adapter sequences, poly-N-containing reads, and low-quality reads. Clean reads were assessed based on their Q20, Q30, and GC contents and aligned to the *Oryza sativa* IRGSP-1.0 top-level reference genome assembly from Ensembl Plants release 57 using HISAT2 (version 2.2.1). The reference genome FASTA file used for read alignment was *Oryza*_*sativa*. IRGSP-1.0.dna.toplevel.fa.gz. Transcript assembly and expression quantification were performed using StringTie (version 2.2.3), and gene-level read counts were obtained using featureCounts (version 2.0.6), with the corresponding Ensembl Plants release 57 gene annotation file, *Oryza*_*sativa*. IRGSP-1.0.57.gff3.gz. The reference genome and annotation files were downloaded from the Ensembl Plants FTP repository. Genome FASTA: https://ftp.ensemblgenomes.ebi.ac.uk/pub/plants/release-57/fasta/oryza_sativa/dna/Oryza_sativa.IRGSP-1.0.dna.toplevel.fa.gz, accessed on 25 May 2025. Gene annotation: https://ftp.ensemblgenomes.ebi.ac.uk/pub/plants/release-57/gff3/oryza_sativa/Oryza_sativa.IRGSP-1.0.57.gff3.gz, accessed on 25 May 2025. FPKM values were used for expression visualization, whereas raw read counts were used for differential expression analysis.

DEGs were identified using DESeq2 (R package version 1.42.0). Genes with a |log2 fold change| ≥ 1 and an adjusted *p*-value < 0.05 were considered differentially expressed, with *p*-values adjusted using the Benjamini–Hochberg method. KEGG pathway enrichment was performed using the clusterProfiler R package, with the detected expressed genes used as the background. Enrichment *p*-values were adjusted using the Benjamini–Hochberg method, and pathways with an adjusted *p*-value < 0.05 were considered significant.

Weighted gene co-expression network analysis was conducted using WGCNA (version 1.71) based on the FPKM matrix. A soft-thresholding power of 14 was selected because it achieved a scale-free topology fit of R^2^ > 0.85, while maintaining moderate mean connectivity and a left-skewed connectivity distribution. Co-expression modules were constructed, and module–trait relationships were assessed using module eigengenes. Highly connected genes in relevant modules were regarded as candidate hub genes.

### 4.5. SRBSDV Infection Assay

The SRBSDV infection assay was conducted as described [31]. Briefly, the virus-free *Sogatella furcifera* (white-backed planthopper) nymphs were allowed to feed on SRBSDV-infected rice seedlings for 3–5 dpi, after which they were transferred to healthy rice plants for an additional 10–12 dpi. Subsequently, SRBSDV-infected/virus-free WBPH were inoculated onto two-week-old rice seedlings (at a rice seedling/insect ratio of 1:3) for 3 dpi. After 30 dpi, the disease symptoms of the plants were examined. After taking photos, all diseased plants were divided into three independent biological replicates. Each biological replicate consisted of tissue pooled from five plants of the same genotype. The pooled tissue was divided for RNA extraction and protein extraction and was therefore used for both RT-qPCR and Western blotting analyses. Each pooled sample was treated as one independent biological replicate. The primers used are listed in Table S6.

For Western blot analysis, total protein was extracted using lysis buffer (100 mM Tris–HCl, pH 6.8, 6% SDS) and centrifuged at 12,000× *g*. The proteins were then separated by 12% SDS-PAGE gel electrophoresis, transferred to a PVDF membrane, and detected using polyclonal anti-P10 antibody (diluted 1:3000) and anti-Actin antibody (diluted 1:5000). Gray values of Western blot bands were quantified with ImageJ.

### 4.6. Data Analysis

For difference analysis, Student’s *t*-test was used when comparing two groups, and one-way ANOVA with Fisher’s least significant difference test was applied when comparing three or more conditions. A *p*-value < 0.05 was considered statistically significant.

## 5. Conclusions

Collectively, our results demonstrate that *OsNOMT* is an important regulator of SAK accumulation and rice resistance to both *M. oryzae* and SRBSDV. Loss of *OsNOMT* abolished SAK accumulation and was associated with increased disease susceptibility, whereas *OsNOMT* overexpression increased the SAK level and enhanced resistance to both pathogens. Transcriptome analysis further revealed that altered *OsNOMT* expression was accompanied by changes in defense-related transcriptional programs, including co-expression modules containing *OsWRKY62*, *OsFBX148*, and a putative ACC oxidase.

Our results also indicate that the *OsNOMT*–SAK module is associated with the regulation of JA metabolism and defense responses. *OsNOMT* overexpression increased JA accumulation and the expression of JA-related defense genes, whereas JA-Ile did not change in parallel with JA. These results suggest that *OsNOMT*-dependent SAK accumulation is linked to a reconfiguration of JA homeostasis rather than a uniform increase in all JA metabolites. Thus, *OsNOMT* may contribute to rice immunity through both the antimicrobial activity of SAK and broader metabolic–hormonal interactions that modulate defense-associated transcription.

This study establishes *OsNOMT* and SAK metabolism as potential targets for improving broad-spectrum disease resistance in rice. However, whether JA signaling is directly required for the *OsNOMT*-dependent resistance phenotypes, and how SAK interacts with specific JA signaling components, remains to be determined through further genetic and biochemical studies. These findings provide a basis for future breeding and biotechnological strategies aimed at developing rice varieties with enhanced resistance to fungal and viral diseases.

## Data Availability

The raw RNA-seq data have been deposited in the National Center for Biotechnology Information (https://www.ncbi.nlm.nih.gov/, accessed on 8 October 2026) under BioProject accession number PRJNA1541158. Further inquiries can be directed to the corresponding authors.

## Supplementary Materials

The following supporting information can be downloaded at: https://www.mdpi.com/article/10.3390/plants15213259/s1, Figure S1: Measurement of OsNOMT-GFP protein level in *OsNOMT-OE* lines by Western blot. Figure S2: KEGG analysis of genes from 10 k-means distinct modules; Figure S3: Venn and KEGG analyses of DEGS in the *OsNOMT-OE*CK_vs_ZH11CK_up and *OsNOMT-OE*MO_vs_*OsNOMT-OE*MO_up groups. Figure S4: Scatter plots of module membership (kME) versus gene significance for key co-expression modules. Figure S5: KEGG analysis of genes from the module membership-gene significance scatter plot analysis; Figure S6: Effect of SAK treatment on the JA pathway. Table S1. KEGG pathway analysis using K-means clustering of genes; Table S2. The identified differentially expressed genes in *OsNOMT-OE*, *osnomt,* and ZH11 plants infected with M. oryzae or not. Table S3. Genes regulated by the three prominent hub genes. Table S4. KEGG pathway analysis using the differentially expressed genes; Table S5. Transcriptome sequencing quality assessment. Table S6. The primers used in this work.
