# Supplementary material for: OsNOMT Contributes to Rice Blast Resistance and Viral Defense and Is Associated with Altered Jasmonate Homeostasis
Source: Plants (Basel). 2026 Oct 22;15(21):3259. doi: 10.3390/plants15213259 (PMC13765950; doi:10.3390/plants15213259)
Supplement: Supplementary file 1 [file plants-15-03259-s001.zip › OsNOMT SI.pdf]

Support information for

OsNOMT contributes to rice blast resistance and viral defense and is associated with altered jasmonate homeostasis

Li Jiang, Sisi He, Xuanyu He, Sifan Wang, Linying Li, Xueying Zhang, Guoyan Zhao, Dali Zeng, Gaojie Hong*, Yuqing He*

#These authors contributed equally to this work

* Corresponding author:

Gaojie Hong, gjhong@126.com

Yuqing He, yqhe_123@126.com

This file includes:

Supplementary Figures 1-6.

.

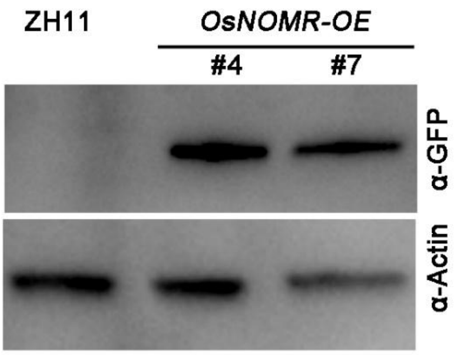

Figure S1. Measurement of OsNOMT-GFP protein level in *OsNOMT-OE* lines by western blot. The Actin was used as control. healthy soil CK

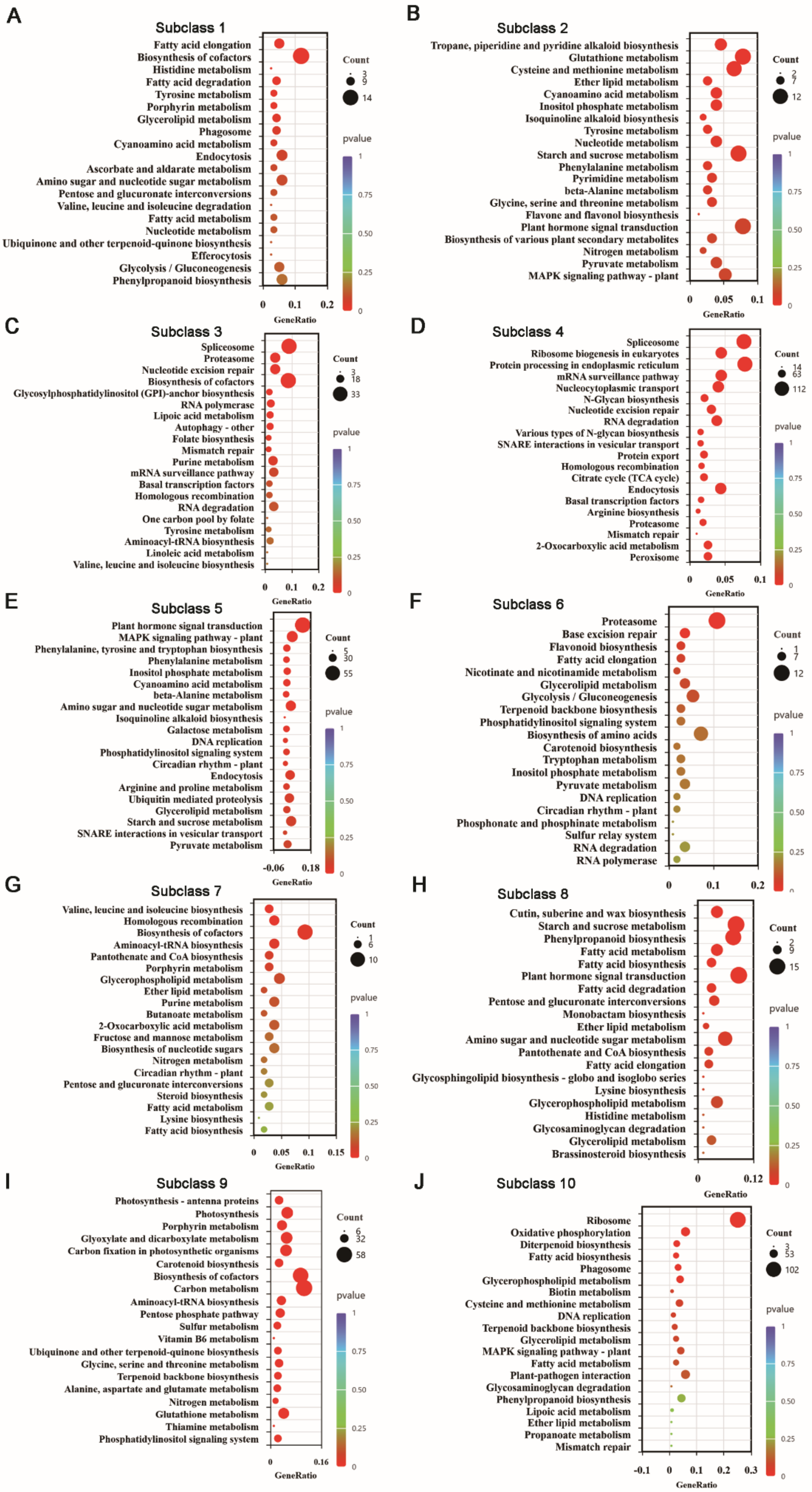

Figure S2. KEGG analysis of genes from 10 k-means distinct modules.

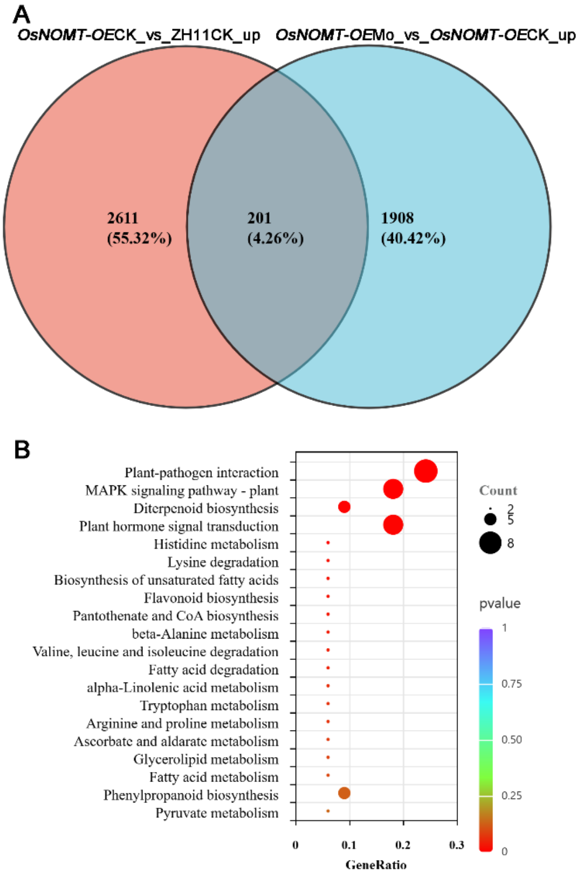

Figure S3. Venn and KEGG analysis of DEGs on *OsNOMT-OE*CK_vs_ZH11CK_up and *OsNOMT-OE*MO_vs_*OsNOMT-OE*CK_up group.

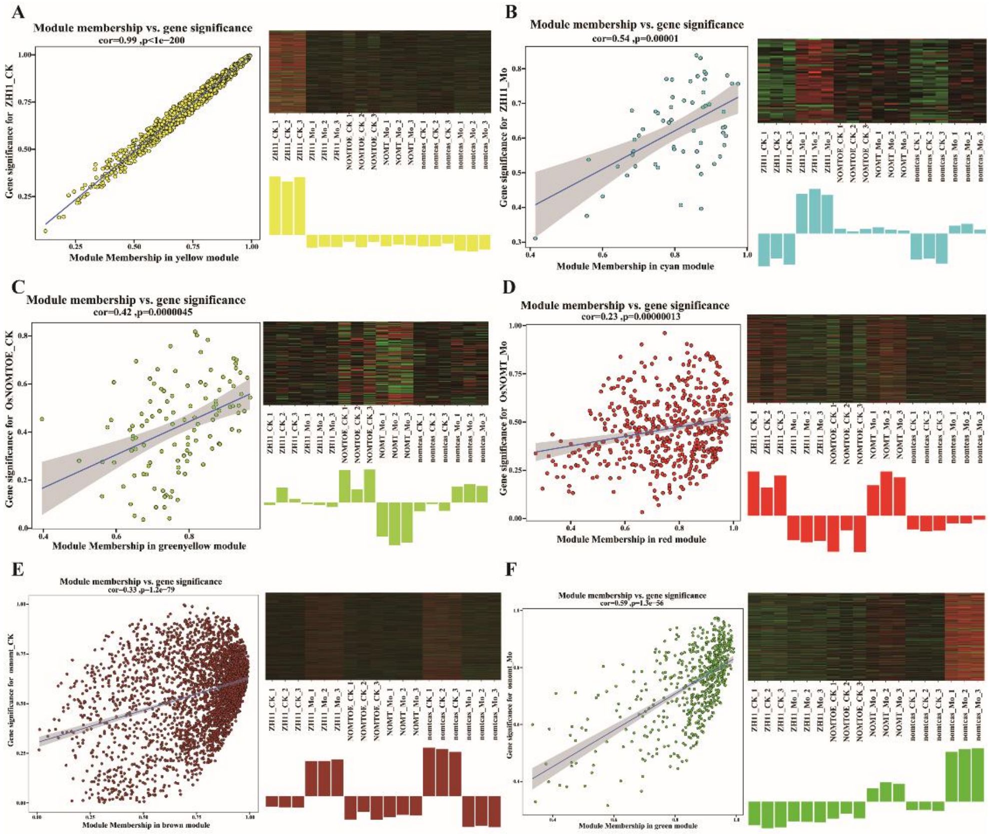

Figure S4. Scatter plots of module membership (kME) versus gene significance for key co-expression modules. **(A)** MEyellow module: highly correlated with ZH11CK sample group (r = 0.99, *p* = 4e-14). **(B)** MEcyan module: highly correlated with ZH11Mo sample group (r = 0.97, *p* = 3.9e-11). **(C)** MEgreenyellow module: highly correlated with *OsNOMT-OE*CK sample group (r = 0.72, *p* = 0.00079). **(D)** MEcyan module: highly correlated with *OsNOMT-OE*Mo sample group (r = 0.57, *p* = 0.014). **(E)** MEbrown module: highly correlated with *osnomt*CK sample group (r = 0.63, *p* = 0.0047) **(F)** MEgreen module: highly correlated with *osnomt*Mo sample group (r = 0.86, *p* = 6e-6).

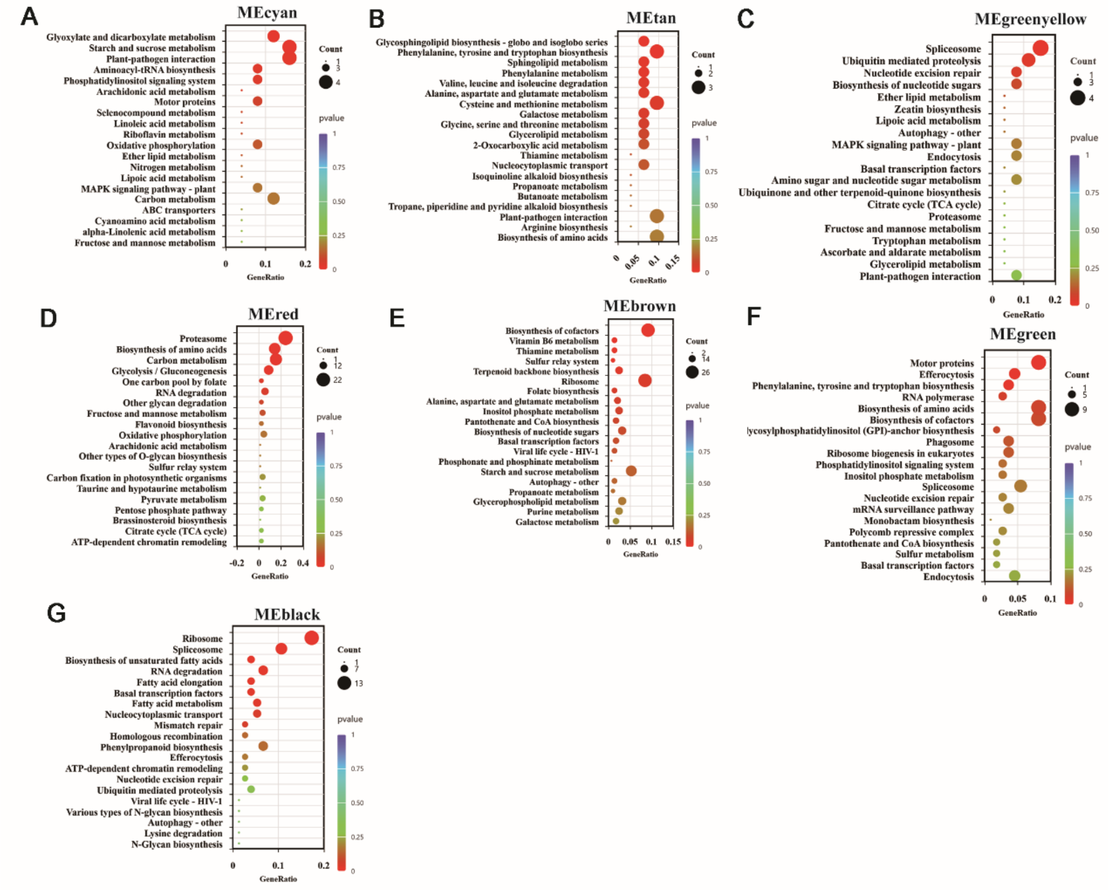

Figure S5. KEGG analysis of genes from module membership-gene significance scatter plots analysis.

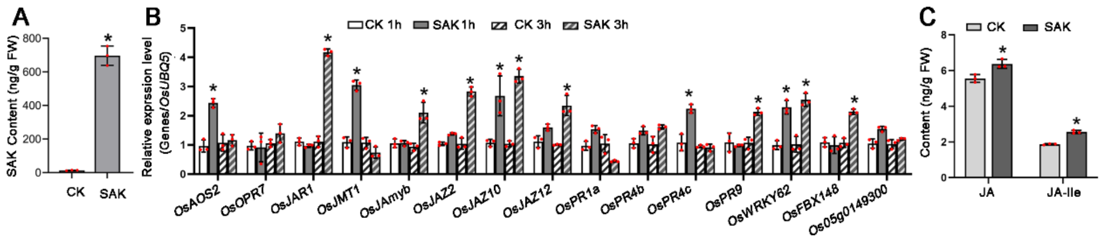

Figure S6. Effect of SAK treatment on JA pathway. (**A**) SAK content after exogenous SAK treatment for 24 h. (**B**) RT-qPCR verification of the JA -related genes. *OsUBQ5* was set as negative control. (**C**) JA and JA-IIe content after SAK treatment for 24 h. Two-week-old ZH11 were sprayed with 0.5 μM SAK at 1 and 3 h for RNA extraction and 24 h for SAK, JA and JA-IIe measurement. Values are mean ± s.d., * $p < 0.05$, Student's *t*-test, $n = 3$.
